# Alterations in the human oral microbiota in systemic lupus erythematosus

**DOI:** 10.1186/s12967-023-03892-3

**Published:** 2023-02-08

**Authors:** Jinyan Guo, Guangying Cui, Wei Huang, Zhaohui Zheng, Tianfang Li, Guanmin Gao, Zhen Huang, Yuwei Zhan, Suying Ding, Shengyun Liu, Zujiang Yu, Zhigang Ren

**Affiliations:** 1 Department of Rheumatology and Immunology, The First Affiliated Hospital of Zhengzhou University, #1 Jianshe East Road, Zhengzhou, 450052 China; 2 Department of Infectious Disease, The First Affiliated Hospital of Zhengzhou University, #1 Jianshe East Road, Zhengzhou, 450052 China; 3 Department of Rheumatology and Immunology, Hainan General Hospital, Haikou, 570100 China; 4 Department of Cardiovascular Disease, The First Affiliated Hospital of Zhengzhou University, Zhengzhou, 450052 China; 5 Health Management Center, The First Affiliated Hospital of Zhengzhou University, Zhengzhou, 450052 China; 6 Gene Hospital of Henan Province, Precision Medicine Center, The First Affiliated Hospital of Zhengzhou University, Zhengzhou, 450052 China; 7 Jinan Microecological Biomedicine Shandong Laboratory, Jinan, 250000 China

**Keywords:** Systemic lupus erythematosus, Oral microbiota, Characteristics, Diagnostic biomarker, Disease activity

## Abstract

**Background:**

Alterations in oral microbiota in patients with systemic lupus erythematosus (SLE) is less evaluated. The aim of this study was to compare the characteristics of the oral microbiome in SLE patients and healthy controls, and construct an SLE classifier based on the oral microbiota.

**Methods:**

We sequenced tongue-coating samples of individuals in treatment-naïve SLE (n = 182) and matched healthy controls (n = 280). We characterized the oral microbiome and constructed a microbial classifier in the derivation cohort and validated the results in the validation cohorts. Furthermore, the oral microbiome of posttreatment SLE (n = 73) was characterized.

**Results:**

The oral microbial diversity of SLE was increased, and the microbial community was different between SLE and healthy controls. The genera Prevotella and Veillonella were enriched, while Streptococcus and Porphyromonas were reduced in SLE. In addition, an increase was noted in 27 predicted microbial functions, while a decrease was noted in 34 other functions. Thirty-nine operational taxonomy units (OTUs) were identified to be related with seven clinical indicators. Two OTUs were identified to construct a classifier, which yielded area under the curve values of 0.9166 (95% CI 0.8848–0.9483, p < 0.0001), 0.8422 (95% CI 0.7687–0.9157, p < 0.0001), and 0.8406 (95% CI 0.7677–0.9135, p < 0.0001) in the derivation, validation, and cross-regional validation groups, respectively. Moreover, as disease activity increased, Abiotrophia and Lactobacillales increased, while Phyllobacterium and unclassified Micrococcusaceae decreased. Finally, nine OTUs were selected to construct a classifier distinguishing posttreatment SLE patients from healthy controls, which achieved a diagnostic efficacy of 0.9942 (95% CI 0.9884–1, p < 0.0001).

**Conclusions:**

Our study comprehensively characterizes the oral microbiome of SLE and shows the potential of the oral microbiota as a non-invasive diagnostic biomarker in SLE.

**Supplementary Information:**

The online version contains supplementary material available at 10.1186/s12967-023-03892-3.

## Introduction

Systemic lupus erythematosus (SLE) is a chronic autoimmune disease featured by loss of autoimmune tolerance and activation of autoimmune response, ultimately resulting in the formation of multiple autoantibodies and damage to various organ systems [1]. It affects females of childbearing age ten times more than males [2]. The clinical manifestations of SLE are markedly heterogeneous, ranging from mild symptoms to life-threatening conditions [3]. To date, the etiology and pathogenesis of SLE is unclear, and it is believed that genetic, race, immune disorders, hormone, and environmental factors may together contribute to the occurrence of the disease [1].

All the microorganisms that inhabit the human body, including bacteria, fungi, archaea, viruses and protozoa, form the human commensal microbiota [4]. Despite the primary habitat being the gut, thriving microbial populations can be found in areas throughout the body, such as the skin, digestive system, respiratory system, and reproductive system [5]. As the largest microsystem in the human body [6, 7], the gut microbiota is considered to be an important environmental trigger for initiating and promoting the progression of SLE through molecular mimicry, interference with the host metabolism axis, and engaging in type I interferon pathways [8–14]. In SLE, the gut microbiota was disturbed with decreased bacterial diversity and altered flora composition and function [9, 15–17], and the gut microbiota was altered along with disease activity changes [18, 19].

The oral microbiota is also an important microecosystem in the human body that is composed of more than 700 unique bacterial species [20].Oral microbiota dysbiosis has been found in many diseases [21–24], and it has been shown that oral microbiota biomarkers could apply to the diagnosis for rheumatoid arthritis (RA) and COVID-19 [21, 24]. However, only a small number of researches have focused on the characteristics of oral microbiota in SLE, and the sample sizes of existing studies were small [25–27]. We conducted the present study to characterize the oral microbiome profiles in patients with SLE from China. We employed 16S rRNA MiSeq sequencing on tongue coating samples from 255 SLE cases and 280 healthy controls (HC) to profile the unique oral microbiota of SLE, identify the unique oral microbiota biomarkers, and establish an oral microbiota diagnostic model of SLE.

## Materials and methods

### Participants and ethic statement

The design of this work was based on prospective specimen collection followed by a blinded retrospective assessment. It was conducted according to Helsinki Declaration. Patients with SLE were consecutively selected from the outpatient clinic and inpatient ward of the Rheumatology Department of the First Affiliated Hospital of Zhengzhou University as well as the Rheumatology Department of Hainan General Hospital between July 2019 and July 2021. Additionally, we included healthy volunteers who went to the First Affiliated Hospital of Zhengzhou University for annual physical examination during the same period.

Included patients satisfied the following criteria: met the classification criteria of SLE in the American College of Rheumatology [28]; aged 18 years or old; did not receive glucocorticoids or any immunosuppressive drugs (treatment-naïve patients), or in stable disease condition under treatment with glucocorticoids (≤ 10 mg/d prednisolone or equivalent) and/or hydroxychloroquine (posttreatment stable patients). The exclusion criteria for SLE were either of the following: complicated with other autoimmune diseases, diabetes, neuropsychiatric diseases, infectious diseases, oral mucosal diseases, gingival and throat diseases; taken antibiotics, probiotics, yogurt, black tea, pickles or other fermented foods in the past 4 weeks; or lacked of clinical data. The collected data of participants included age, gender, body mass index (BMI), routine blood and urine tests, HBsAg, HBsAb, anti-HCV, TPPA, HIV antibody, liver function, kidney function. Data of antinuclear antibody, anti- extractable nuclear antigen antibody, complement C3 and C4, immunoglobulins A, G and M were collected from SLE patients. The initial protocol of the study obtained approval of the Ethics Committee at the First Affiliated Hospital of Zhengzhou University (2019-KY-200). All participants signed a written informed consent prior to participation.

### Sample collection

All subjects gargled with sterile mouthwash twice before collecting tongue coating samples, and then, professional operators scraped the posterior to anterior middle regions of the tongue coating with swabs. The collected tongue coating specimen was immediately put into the tube and transferred to a − 80 °C refrigerator within 2 h.

### DNA extraction, PCR amplification, and sequencing

Extraction of bacterial DNA was done via the E.Z.N.A. ® Stool DNA Kit (Omega Bio-tek, Inc., GA) as per the steps stated in the kit’s instructions. Primers targeting the V3-V4 region (5ʹ- CCTACGGGNGGCWGCAG -3ʹ and 5ʹ-GACTACHVGGGTATCTAATCC-3ʹ) with high variation of 16S rRNA were used for PCR amplification of the extracted DNA samples. DNA sequences were identified on an Illumina MiSeq platform (Illumina Inc., USA) by Shanghai Mobio Biomedical Technology Co., Ltd. Raw data of the collected samples were stored in the Sequence Read Archive database (PRJNA789129).

### Bioinformatics

USEARCH (version 11.0.667) was used to process raw sequencing data. UPARSE (http://drive5.com/uparse/) was used to classify operational taxonomy units (OTUs) based on 97% sequence similarity, and OTUs were annotated using the SILVA reference database (SSU138). The Shannon, observed OTUs, Ace, Chao and Simpson indices were calculated by using Mothur v1.42.1 to evaluate α diversity. The R package (version 3.6.0) was used to analyze β diversity of bacteria, including nonmetric multidimensional scaling (NMDS), principal coordinate analysis (PCoA) and principal component analysis (PCA).

As per the normalized relative abundance matrix, linear discriminant analysis (LDA) and LDA effect size (LEfSe) were adopted (https://github.com/SegataLab/lefse) to analyze the characteristics between different groups of bacteria. The threshold was set to LDA score (log10) = 3. PICRUSt2 v2.4.1 (https://github.com/picrust/picrust2/wiki) was employed to predict functional abundances based on 16S rRNA gene sequences.

By verifying the reads in the representative sequence of the derivation and the validation sets, the OTU frequencies of the derivation and the validation sets are obtained. Using the Wilcoxon test (p < 0.05), significant OTU biomarkers were isolated to undergo further analyses. The identified OTUs from the derivation set was used for construct diagnostic model based on random forest model. To obtain the optimal combination of diagnostic markers, we ranked OTUs from high to low according to their importance values (Mean Decrease Accuracy), increased the number of OTUs in order, and calculated the error of constructing the diagnostic model using different numbers of biomarkers. Five-fold cross-validation was also performed in the derivation set to obtain five different cross-validation (CV) error curves. The mean value of CV error is calculated based on the data of the five curves, and a line graph drawn with the mean value is obtained. We select the minimum CV-error in the mean curve plus the standard deviation of the CV error as the cut-off value. We list all sets of OTU markers whose CV error is less than the cut-off value and select the set with the smallest number of OTUs as the optimal set. Finally, the probability of disease (POD) index of the derivation and validation sets were calculated by the determined optimal OTU set. The POD index refers to the ratio of the number of randomly generated decision trees whose predicted sample is SLE to to the number of predicted HC [24, 29]. Then, a receiver operating characteristic (ROC) curve (R 3.3.0, pROC package) was constructed to evaluated the model through the area under the curve (AUC).

### Statistical analysis

Continuous variables that are normally and non-normal distributed were represented by the mean (standard deviation) and median (inter-quartile range), respectively. Categorical variables were expressed as percentages. To compare the difference between SLE patients and HC, Student's t-test and Wilcoxon rank-sum test were used for continuous variables with normal and non-normal distribution, respectively. In addition, the Chi-square test or Fisher's exact test was used for categorical variables. SPSS V.21.0 (SPSS, Chicago, Illinois, USA) was used to conduct the statistical analysis. Differences were identified as statistically significant with p < 0.05.

## Results

### Study design and characteristics of the participants

**Fig. 1:**
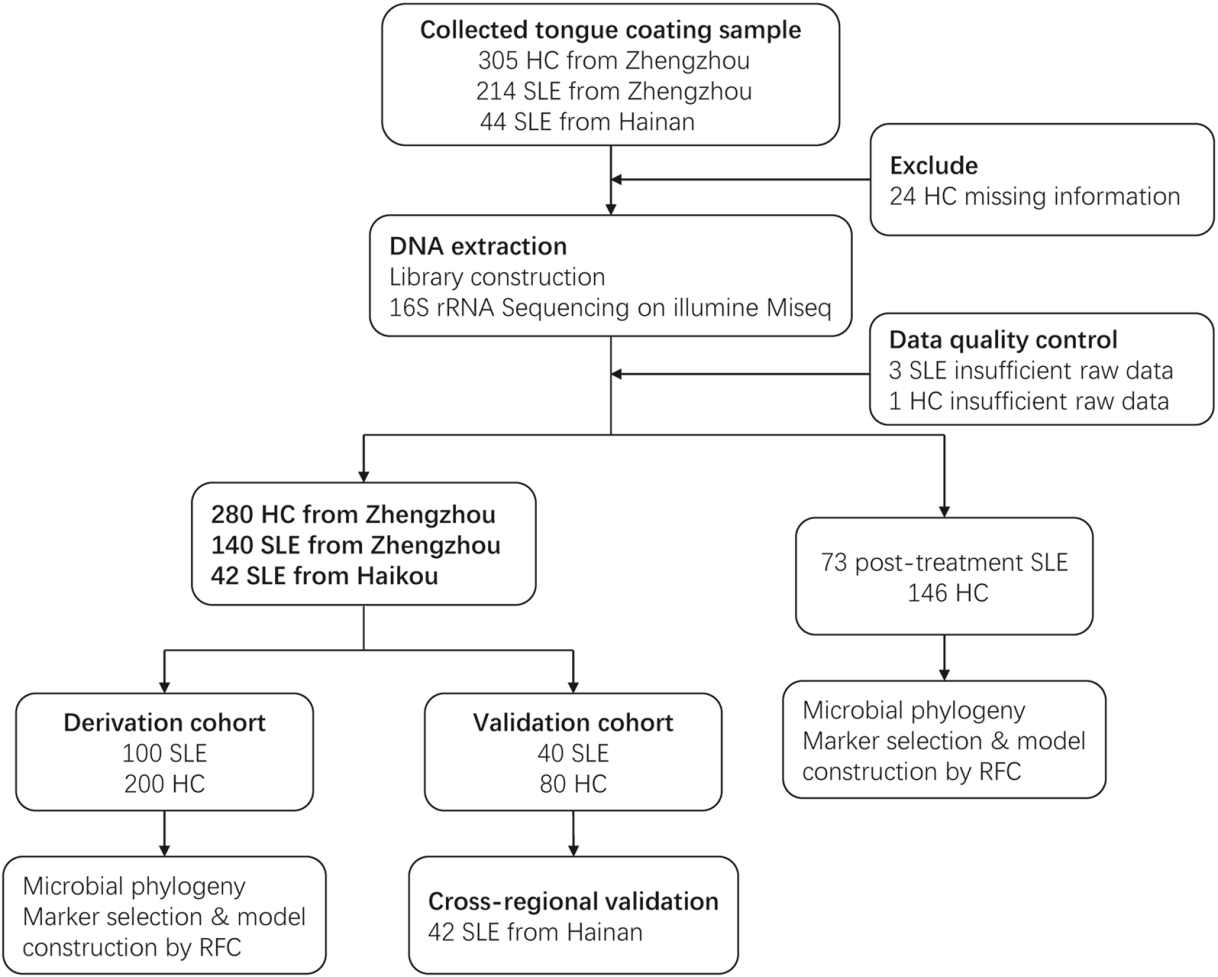
Study design and flow diagram. A total of 563 tongue coating samples from different regions of China were collected prospectively. After rigorous diagnosis and exclusion procedures, 535 tongue coating samples were included for analysis, including 213 SLE (140 treatment-naïve patients and 73 posttreatment patients) from Zhengzhou, 280 HC from Zhengzhou, and 42 treatment-naïve SLE patients from Haikou. The treatment-naïve samples from Zhengzhou were randomly divided into a derivation cohort and a validation cohort based on a random number table. The derivation cohort was used to characterize the oral microbiome of SLE, identify key microbial markers, and constructed an SLE classifier. The validation and cross-regional validation cohorts were used to evaluate the diagnostic efficacy of the classifier. We further characterized the oral microbiome of 73 posttreatment SLE patients and 146 HC. *HC* healthy controls, *SLE* systemic lupus erythematosus, *RFC* random forest classifier model

An overall number of 563 tongue coating samples were collected from variable areas around China prospectively. After strict exclusion and diagnosis procedures, 535 tongue coating samples were analyzed, including 213 SLE (140 treatment-naïve patients and 73 posttreatment patients) from Zhengzhou, 280 HC from Zhengzhou, and 42 treatment-naïve SLE patients from Haikou. The treatment-naïve samples from Zhengzhou were randomly divided into a derivation cohort (100 SLE vs. 200 HC) and an independent validation cohort (40 SLE vs. 80 HC) based on a random number table. Characterization of the oral microbiome, identification of the key microbial markers, and construction of an SLE classifier were performed in the derivation cohort. The efficacy of diagnosis of the SLE classifier was validated in the independent validation and cross-regional validation cohort (42 SLE from Haikou vs. 80 HC). We further divided the 140 SLE patients from Zhengzhou into three groups according to the SLE disease activity index (SLEDAI) and characterized the oral microbiome in these three groups [30, 31]. Finally, we compared the oral microbiome of 73 posttreatment SLE patients and 146 HC (Fig. 1).

In the derivation and validation cohorts, the genders, ages, and BMI of SLE and HC were matched. Serum levels of white blood cells, red blood cells, hemoglobin, platelets, and lymphocyte counts were decreased significantly, and serum levels of globulin were increased significantly in SLE compared with HC. The detail is shown in Table 1.

**Table 1:** Clinical characteristics of the participants

Clinical indices	Derivation		P value^a^	Validation		P value^a^	P value^b^
	SLE (n = 100)	HCs (n = 200)		SLE (n = 40)	HCs (n = 80)		
Age	34 (25, 41.75)	32 (28, 38.75)	0.1004	33 (27, 43)	31.5 (28, 35)	0.3096	0.7533
Gender (F/M)	94/6	184/16	0.5310	37/3	75/5	0.7958	0.7437
BMI (Kg/m^2^)	21.81 (19.50, 24.13)	22.05 (20.88, 23.16)	0.9682	21.10 (19.08, 24.59)	21.94 (21.03, 23.22)	0.0626	0.1836
WBC (× 10^9^/L)	5.08 (3.98, 6.72)	5.90 (5.10, 7.10)	< 0.0001	4.78 (3.67, 6.85)	6.40 (5.3, 7.4)	0.0009	0.7711
RBC (× 10^12^/L)	4.02 ± 0.53	4.71 ± 0.49	< 0.0001	3.99 ± 0.64	4.78 ± 0.44	< 0.0001	0.8553
Hb (g/L)	120.2 ± 17.81	138.2 ± 16.02	< 0.0001	118.2 ± 19.95	145.8 ± 13.8	< 0.0001	0.6617
Plt (× 10^9^/L)	201 (162.8, 257.8)	235 (201.5, 265)	0.0002	180 (127, 256)	241 (212, 269)	0.0006	0.2520
Lyn# (× 10^9^/L)	1.23 (0.85, 1.56)	1.99 (1.66, 2.38)	< 0.0001	1.31 (0.85, 1.82)	2.07 (1.84, 2.49)	< 0.0001	0.4767
Glb (g/L)	29.6 (26.5, 33.7)	26.8 (24.5, 28.9)	< 0.0001	31.45 (26.15, 35.73)	26.10 (24.05, 28.65)	< 0.0001	0.4415
C3 (0.9–1.8 g/L)	0.82 (0.66, 1.01)	–	–	0.82 (0.71, 1.10)	–	–	0.3943
C4 (0.1–0.4 g/L)	0.14 (0.11, 0.20)	–	–	0.15 (0.11, 0.20)	–	–	0.9328
Anti-dsDNA (0–100 IU/mL)	198 (15.48, 779)	–	–	120.9 (16.25, 686)	–	–	0.7627
Disease activity							
Mild/moderate/severe	50/41/9	–	–	18/17/5	–	–	0.5190

*SLE* systemic lupus erythematosus, *HCs* health control, *F* female, *M* male, *BMI* body mass index, *WBC* white blood cell count, *RBC* red blood cell count, *Hb* hemoglobin, *Plt* platelet count, *Lyn#* lymphocyte count, *Glb* globulin, *C3* complement C3, *C4* complement C4

^a^Comparisons between SLE and HCs

^b^Comparisons between SLE in the derivation cohort and those in the validation cohort

### Increased oral microbial diversity in SLE

**Fig. 2:**
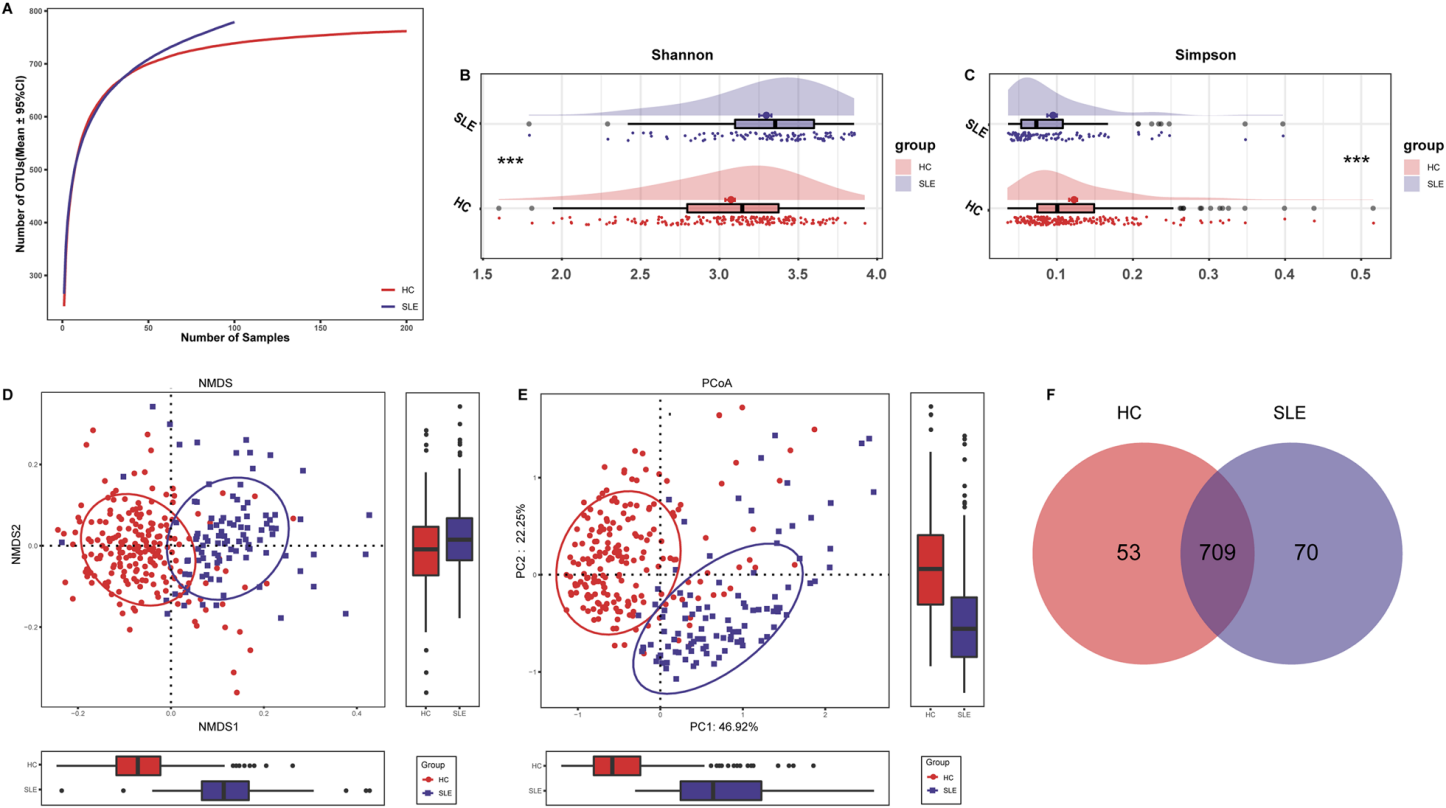
Oral microbial diversity in SLE was increased. **A** Rarefaction analysis between the number of samples and the number of OTUs. As the number of samples increased, the number of OTUs approached saturation in SLE (n = 100) and HC (n = 200). Compared with the HC, the number of OTUs in SLE was increased significantly. **B** As measured by the Shannon index, the oral microbial diversity was significantly increased in SLE (n = 100) versus HC (n = 200) (p < 0.001). **C** As measured by the Simpson index, the oral microbial diversity was significantly increased in SLE (n = 100) versus HC (n = 200) (p < 0.001). **D** As estimated by the NMDS analysis, the OTU distribution was significantly different between SLE (n = 100) and HC (n = 200). **E** As estimated by the PCoA, the OTU distribution was significantly different between SLE (n = 100) and HC (n = 200). **F** A Venn diagram showed that 709 out of 832 OTUs were shared in both groups, while 70 OTUs were specific to SLE (n = 100). ***, p < 0.001. *HC* healthy controls, *SLE* systemic lupus erythematosus, *OTU* operational taxonomic unit, *NMDS* nonmetric multidimensional scaling, *PCoA*, principal coordinate analysis

During the derivation cohort, the analysis of rarefaction revealed that the count of OTU richness almost reached saturation in both groups, and it was increased significantly in SLE versus HC (Fig. 2A). The results of the rarefaction analysis were shown in Additional file 1: Fig. S1. As measured by the Shannon and Simpson indices, the oral microbial diversity increased significantly in SLE compared with HC (all p < 0.001), (Additional file 2: Data S1*,* Fig. 2B, C).

In addition, beta diversity was conducted to display microbiome space between each sample. As estimated by NMDS and PCoA analysis, the OTU distribution differed significantly between the two groups (Fig. 2D, E, Additional file 1: Fig. S2). A Venn diagram illustrated the overlap of OTUs between SLE and HC. Totally, 709 OTUs were shared, while 70 OTUs were specific to SLE (Fig. 2F). The key 46 OTUs between the two groups was shown in Additional file 1: Fig. S3; (Additional file 2: Data S2). Notably, 24 OTUs showed significant enrichment in SLE, and 22 OTUs showed significant enrichment in HC.

### Phylogenetic profiles of the oral microbiome in SLE

**Fig. 3:**
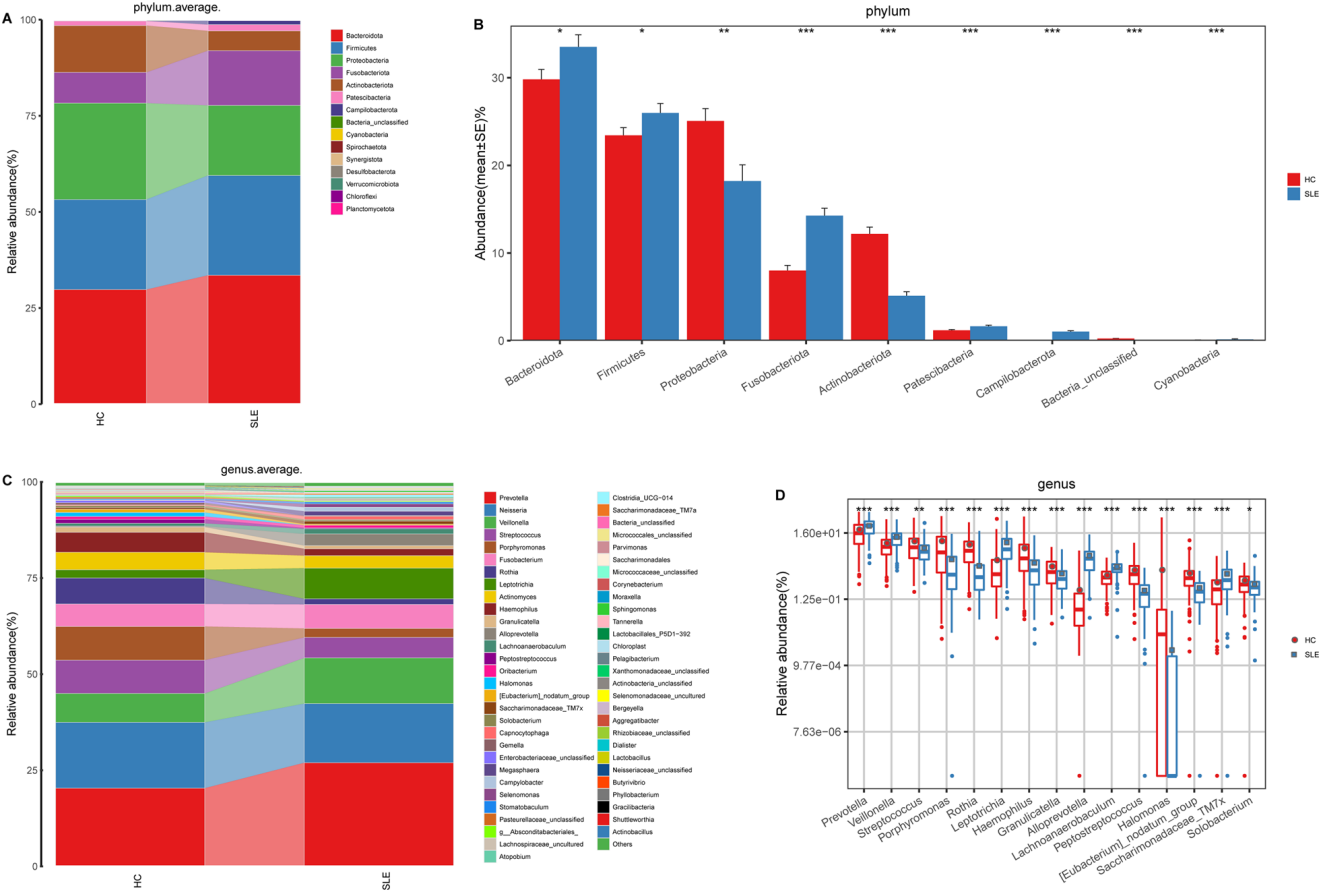
Phylogenetic profiles of the oral microbiome in SLE. **A** The average composition and relative abundance of the bacterial community in both groups at the phylum level. **B** Compared with HC, SLE patients exhibited increased abundance of six phyla and decreased abundance of three phyla. **C** The average composition and relative abundance of the bacterial community in both groups at the genus level. **D** Compared with HC, SLE patients exhibited increased abundance of 28 genera and decreased abundance of 22 genera. *, p < 0.05, **, p < 0.01, ***, p < 0.001. *HC* healthy controls, *SLE* systemic lupus erythematosus

The taxa composition and variation of the oral microbiota were compared in SLE and HC in the derivation cohort. At the phylum and genus levels, the relative abundance of taxa of oral microbiota in each sample were displayed in Additional file 1: Figs. S4 and S5 (Additional file 2: Data S3 and S4), and the average composition and relative abundance of taxa in both groups were shown in Fig. 3A and C, respectively. Bacteroidota, Firmicutes and Proteobacteria were the three most predominant phyla in both groups.

We further analyzed bacterial abundance to identify key differentially abundant bacteria between the two groups. As shown in Fig. 3B, SLE patients exhibited increased abundance of six phyla, including Bacteroidota, Firmicutes, Fusobacteriota, Patescibacteria, Campilobacterota and Cyanobacteria, and decreased abundance of Proteobacteria, Actinobacteriota, and Bacteria_unclassified compared with HC (p < 0.05), (Additional file 2: Data S5). Figure 3D showed that increased abundance in 28 bacteria, including Prevotella, Veillonella, Leptotrichia, Alloprevotella, and Lachnoanaerobaculum, and decreased abundance in 22 bacteria, including Streptococcus, Porphyromonas, Rothia, Haemophilus, and Granulicatella, were observed in SLE at the genus level (p < 0.05) (Additional file 2: Data S6). In addition, we analyzed the oral microbiota community in the two groups at the class, order, and family levels. The composition and abundance of taxa in each sample at the class level were shown in Additional file 1: Fig. S6, while at the order level and at the family level are shown in Additional file 1: Fig. S9 and Figure S12, respectively. At class level, the relative abundance of Bacteroidia and Gammaproteobacteria is relatively high, reaching more than 50% in both HC and SLE (Additional file 1: Fig. S7); at the order level, the relative abundance in the two groups was higher for Bacteroidales, Burkholderiales and Fusobacteriales (Additional file 1: Fig. S10); while at the family level, the relative abundance in the two groups was higher for Prevotellaceae, Neisseriaceae and Veillonellaceae (Additional file 1: Fig. S13). At the class level, compared to HC, SLE had 8 bacterial populations significantly enriched (e.g., Cyanobacteriia, Coriobacteriia, and Campylobacteriia) and 4 bacterial populations significantly reduced (e.g., Actinobacteria, Bacilli and Gammaproteobacteria) (Additional file 1: Fig. S8 and Additional file 2: Data S7); At the order level, SLE had 13 bacterial populations significantly enriched (e.g., Campylobacterales, Flavobacteriales, and Pseudomonadales) and 12 bacterial populations significantly reduced (e.g., Micrococcales, Pasteurellales and Staphylococcales) (Additional file 1: Fig. S11 and Additional file 2: Data S8); while at the family level, SLE had 17 bacterial populations significantly enriched (e.g., Campylobacteraceae, Sphingomonadaceae, and Chloroplast) and 18 bacterial populations significantly reduced (e.g., Micrococcaceae, Pasteurellaceae and Porphyromonadaceae) (Additional file 1: Figs. S14 and Additional file 2: Data S9).

### Crucial bacteria and microbial functions related to SLE

**Fig. 4:**
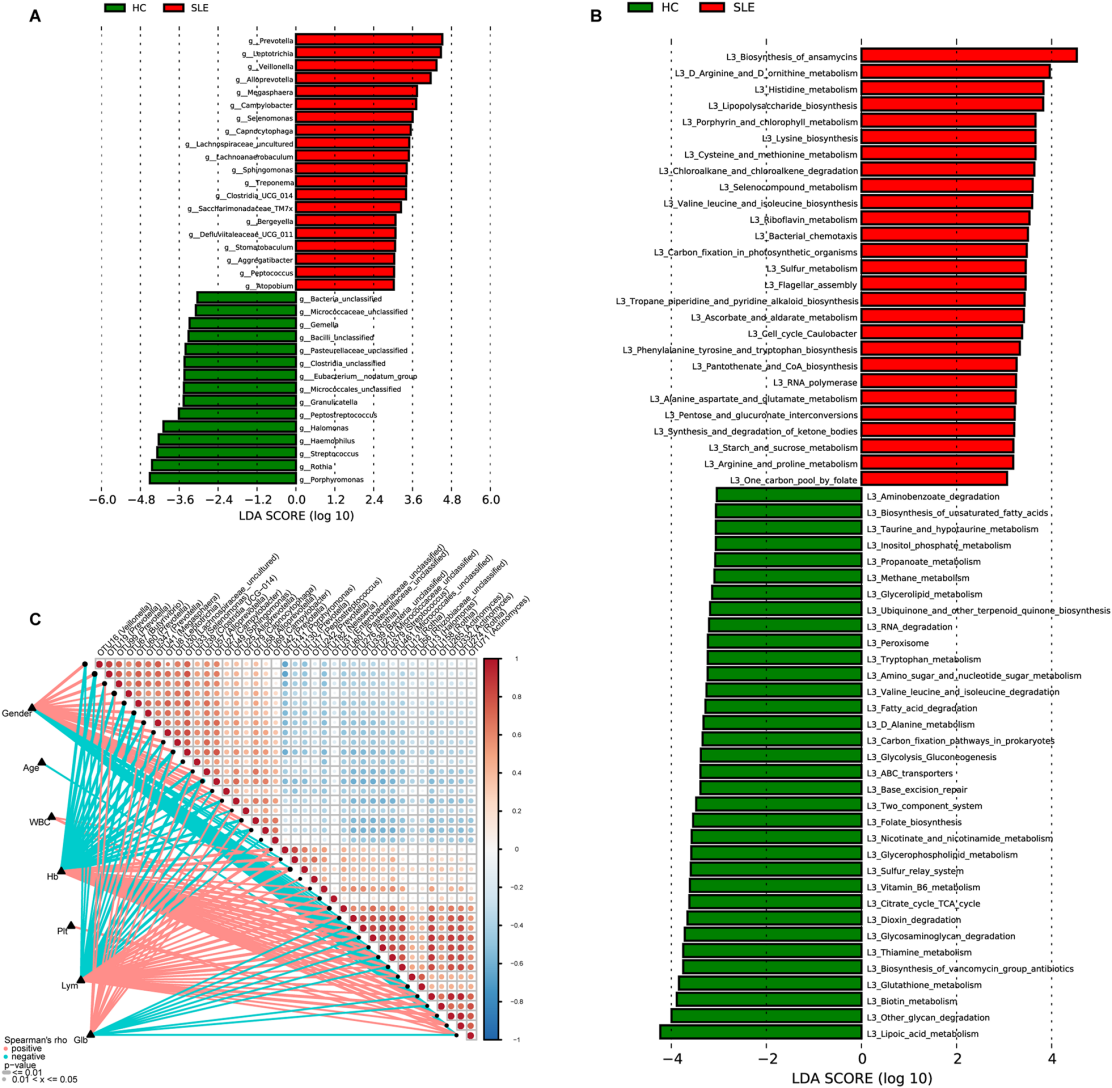
Crucial bacteria of oral microbiome and microbial functions related to SLE. **A** Crucial bacteria of oral microbiome related to SLE. Based on the LDA selection, 20 genera were significantly enriched, while 15 genera were significantly reduced in SLE (n = 100) compared with HC (n = 200) (all p < 0.01). **B** Crucial microbial predicted functions related to SLE. Based on the LDA selection, 27 predicted microbial functions were remarkably increased, while 34 functions were remarkably decreased in SLE (n = 100) compared with HC (n = 200) (all p < 0.05). **C** Distance correlation plots of relative abundances of 39 OTUs and the clinical indices Gender, Age, WBC, Hb, Plt, Lym, and Glb. *HC* healthy controls, *SLE* systemic lupus erythematosus, *LDA* linear discriminant analysis, *WBC* white blood cell, *Hb* hemoglobin, *Plt* platelet count, *Lym* lymphocyte count, *Glb* globulin

To identify the special bacterial and key bacterial taxa contributing to the alteration of SLE oral microbiota, we performed LDA and LEfSe to identify the difference between SLE and HC. A cladogram revealing the phylogenetic profile of different bacterial taxa showed the maximum differences in taxa between the two groups, which implied dysbiosis of oral microbiota in SLE (Additional file 1: Fig. S15, Additional file 2: Data S10). At the genus level, considerable differences were observed between SLE and HC according to LDA selection. As shown in Fig. 4A, increased abundance in 20 genera, including Prevotella, Leptotrichia, Veillonella, Alloprevotella, and Megasphaera, and decreased abundance in 15 bacteria, including Bacteria_unclassified, Micrococcaceae_unclassified, Gemella, Bacilli_unclassified, and Pasteurellaceae_unclassified, were observed in SLE compared with HC (p < 0.01) (Additional file 2: Data S11).

The functional profiles and the key microbial functions between two groups were shown in Additional file 1: Fig. S16, which implied dysbiosis of functional patterns in SLE. Compared with HC, 27 functional modules, including ansamycin biosynthesis, arginine and ornithine metabolism, and histidine metabolism increased significantly, while 34 functions, including aminobenzoate degradation, unsaturated fatty acid biosynthesis, and taurine and hypotaurine metabolisms decreased significantly in SLE (all p < 0.05), (Fig. 4B, Additional file 2: Data S12). We further analyzed the correlations between oral microbiome and clinical data of SLE by Spearman’s correlation analysis. As shown in Fig. 4C and Additional file 1: Fig. S17. 39 OTUs were related to seven clinical indicators, including gender, age, white blood cell count, hemoglobin, platelet count, lymphocyte count, and globulin (Additional file 2: Data S13).

### Diagnostic potential of the oral microbial markers for SLE

**Fig. 5:**
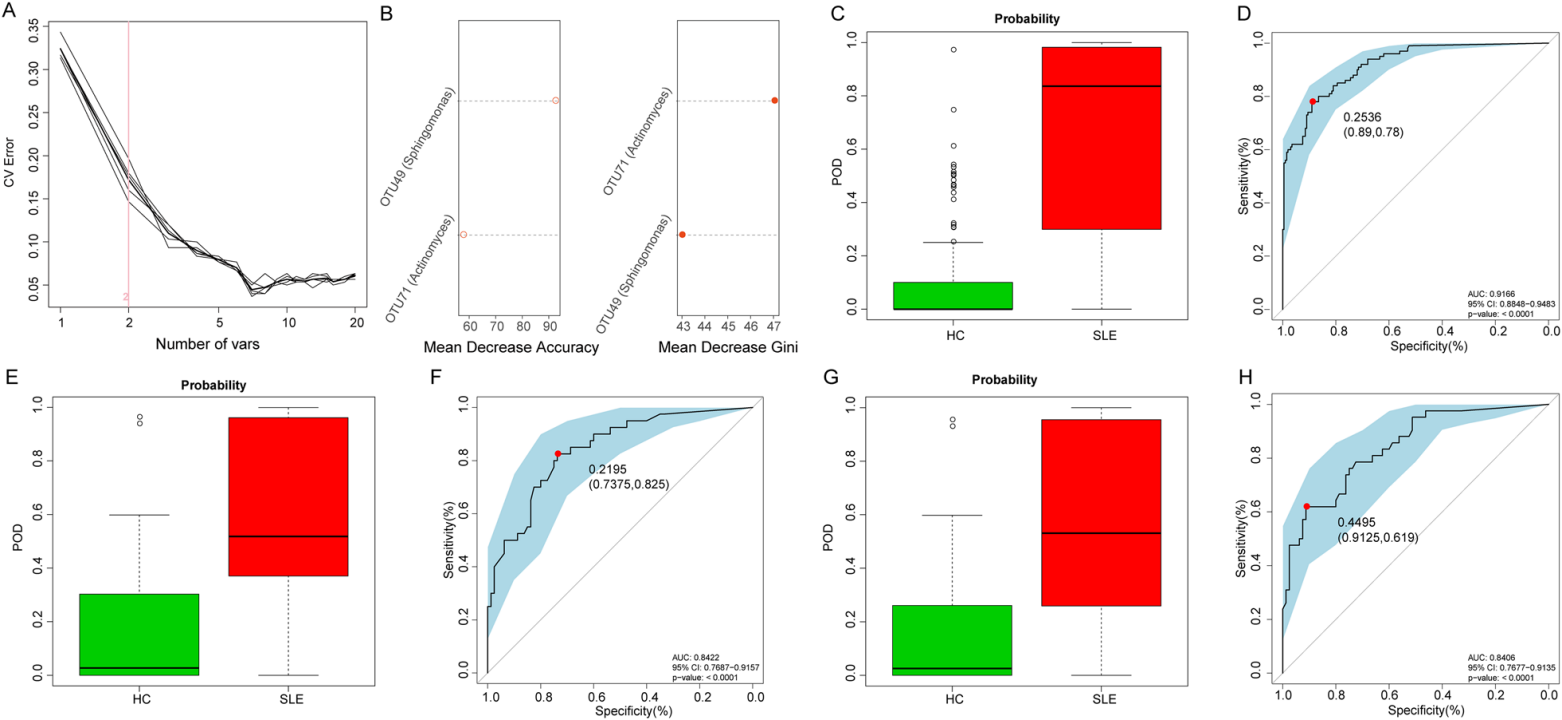
Diagnostic potential of oral microbial markers for SLE. **A** Two bacterial markers were selected as the best markers set by random forest model through five-fold cross-validation. **B** Importance distribution map of the selected microbial markers in the model. **C** The POD value was significantly higher in SLE (n = 100) compared with that in HC (n = 200) in the derivation cohort. **D** The POD value achieved an AUC of 0.9166 (95% CI 0.8848–0.9483) between SLE (n = 100) versus HC (n = 200) in the derivation cohort (p < 0.0001). **E** The POD value was significantly higher in SLE (n = 40) than that in HC (n = 80) in the independent validation cohort (p < 0.05). **F** The POD value achieved an AUC of 0.8422 (95% CI 0.7687–0.9157) between SLE (n = 40) versus HC (n = 80) in the independent validation cohort (p < 0.0001). **G** The POD value was significantly higher in SLE (n = 42) than that in HC (n = 80) in the cross-reginal validation cohort (p < 0.05). **H** The POD value achieved an AUC of 0.8406 (95% CI 0.7677–0.9135) between SLE (n = 42) versus HC (n = 80) in the cross-reginal validation cohort (p < 0.0001). CV Error, cross-validation error; *HC* healthy controls, *SLE* systemic lupus erythematosus; *POD* probability of disease, *OUT* operational taxonomic unit, *CI* confidence interval, *AUC* area under the curve

For assessment of the diagnostic potential of oral microbial markers in SLE, a random forest classifier model was established in order to distinguish SLE from HC in the derivation group. A total of two OTU markers, including OTU49 (Sphingomonas) and OTU71 (Actinomyces) were identified as the optimal marker set (Fig. 5A, B). The relative abundance of the two OTU markers in each sample was displayed in Additional file 2: Data S14. Then, the two OTUs were used in the calculation of the POD value of each sample in the derivation group (Additional file 2: Data S15). As shown in Fig. 5C and D, the POD value was higher in SLE than in HC (p < 0.05), and it reached an AUC of 0.9166 (95% CI 0.8848–0.9483, p < 0.0001), which implied that the oral microbial marker-based classifier had powerful diagnostic potential for distinguishing SLE from HC.

In addition, an independent validation and a cross-regional validation group were used for validation of the diagnostic efficiency of the microbial marker model for SLE. Additional file 2: Datas S16 and S17 showed the relative abundance of the two OTU markers in each sample in both groups. Figure 5E and G displayed that there was a significant increase in POD value in SLE than in HC in the independent validation and cross-reginal validation groups (all p < 0.05) (Additional file 2: Datas S18 and S19). The AUC value of PODs reached 0.8422 (95% CI 0.7687–0.9157, p < 0.0001) and 0.8406 (95% CI 0.7677–0.9135, p < 0.0001) in the independent validation and cross-regional validation groups (Fig. 5F and H), respectively, which validated the diagnostic potential of the oral microbial marker-based classifier model for SLE.

### Oral microbiome alterations in SLE with different disease activities

**Fig. 6:**
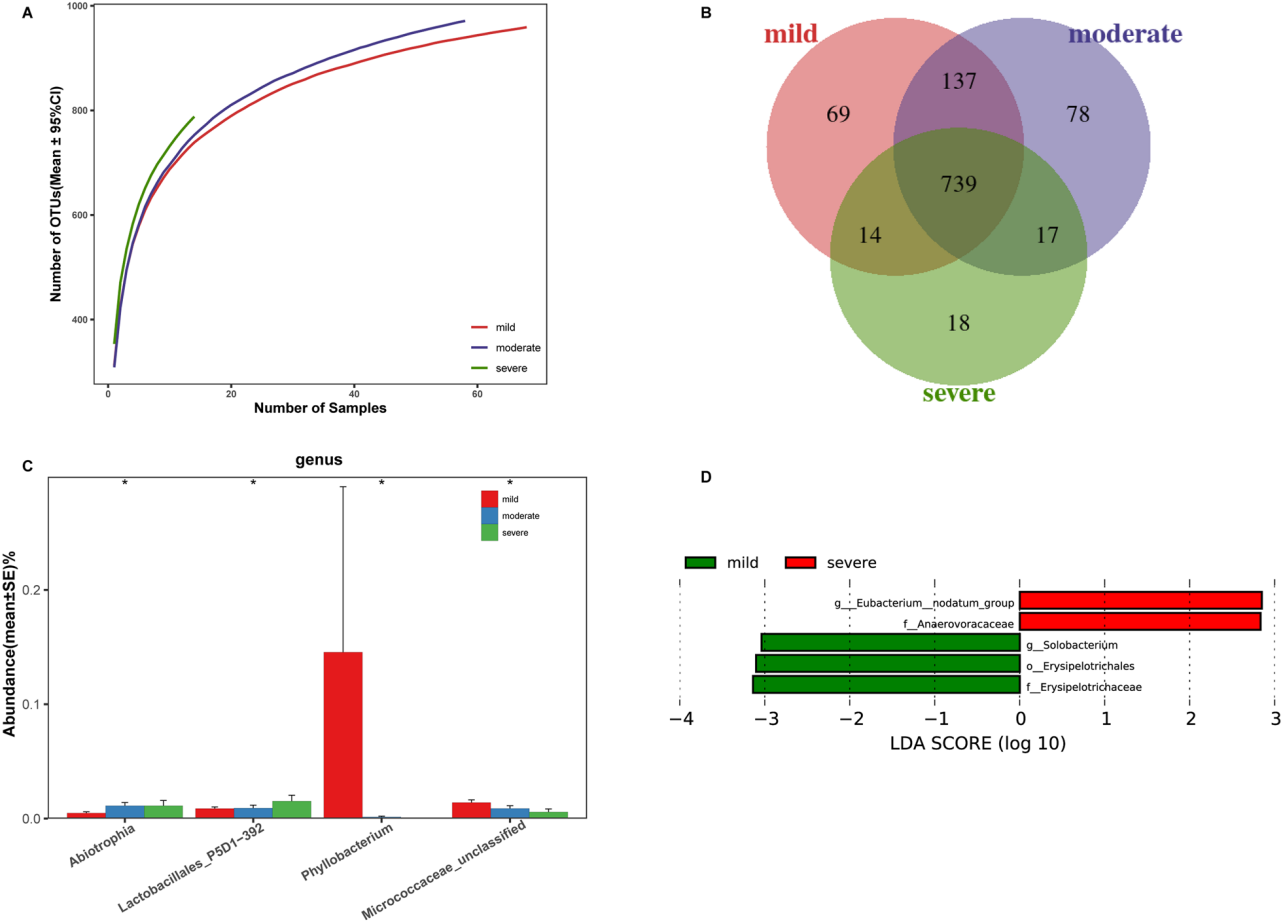
Oral microbiome alterations in SLE with different disease activity. **A** Rarefaction analysis between the number of samples and the number of OTUs. Rarefaction analysis showed that the number of OUT richness was significantly increased as disease activity increased among the three groups. **B** A Venn diagram showed that 69 OTUs, 78 OTUs, and 18 OTUs were unique to mild, moderate, and severe disease activity groups, respectively. **C** At the genus level, Abiotrophia, and Lactobacillales_P5D1-392 were enriched, and Phyllobacterium, and Micrococcaceae_unclassified were decreased as disease activity increasing. **D** Based on the LDA selection, two and three oral microbial taxa were enriched in mild (n = 68) and severe (n = 14) disease activity groups, respectively (all p < 0.05) *, p < 0.05; **, p < 0.01; *OUT* operational taxonomic unit, *LDA* linear discriminant analysis

To specify the characteristics of the oral microbiome in variable disease activities of SLE, the 140 SLE specimens were divided into mild (n = 68, SLEDAI ≤ 6), moderate (n = 58, SLEDAI 7–11), and severe (n = 14, SLEDAI ≥ 12) disease activity groups. Among the three groups, the analysis of rarefaction showed that the number of OTU richness was significantly increased as disease activity increased (Fig. 6A). Moreover, there were 69 OTUs, 78 OTUs, and 18 OTUs unique to the groups with mild, moderate, and severe disease activity, respectively (Fig. 6B). At the genus level, increased abundance in Abiotrophia and Lactobacillales_P5D1-392, and decreased abundance in Phyllobacterium and Micrococcaceae_unclassified were observed as disease activity increased (p < 0.05), (Fig. 6C, Additional file 2: Data S20). Based on LDA analysis, Eubacterium_nodatum_group and Anaerovoracaceae enrichment was significant in the severe disease activity group, while Solobacterium, Erysipelotrichales, and Erysipelotrichaceae enrichment was significant in the mild group (Fig. 6D, Additional file 2: Data S21).

### Alterations of oral microbiota in posttreatment stable SLE

**Fig. 7:**
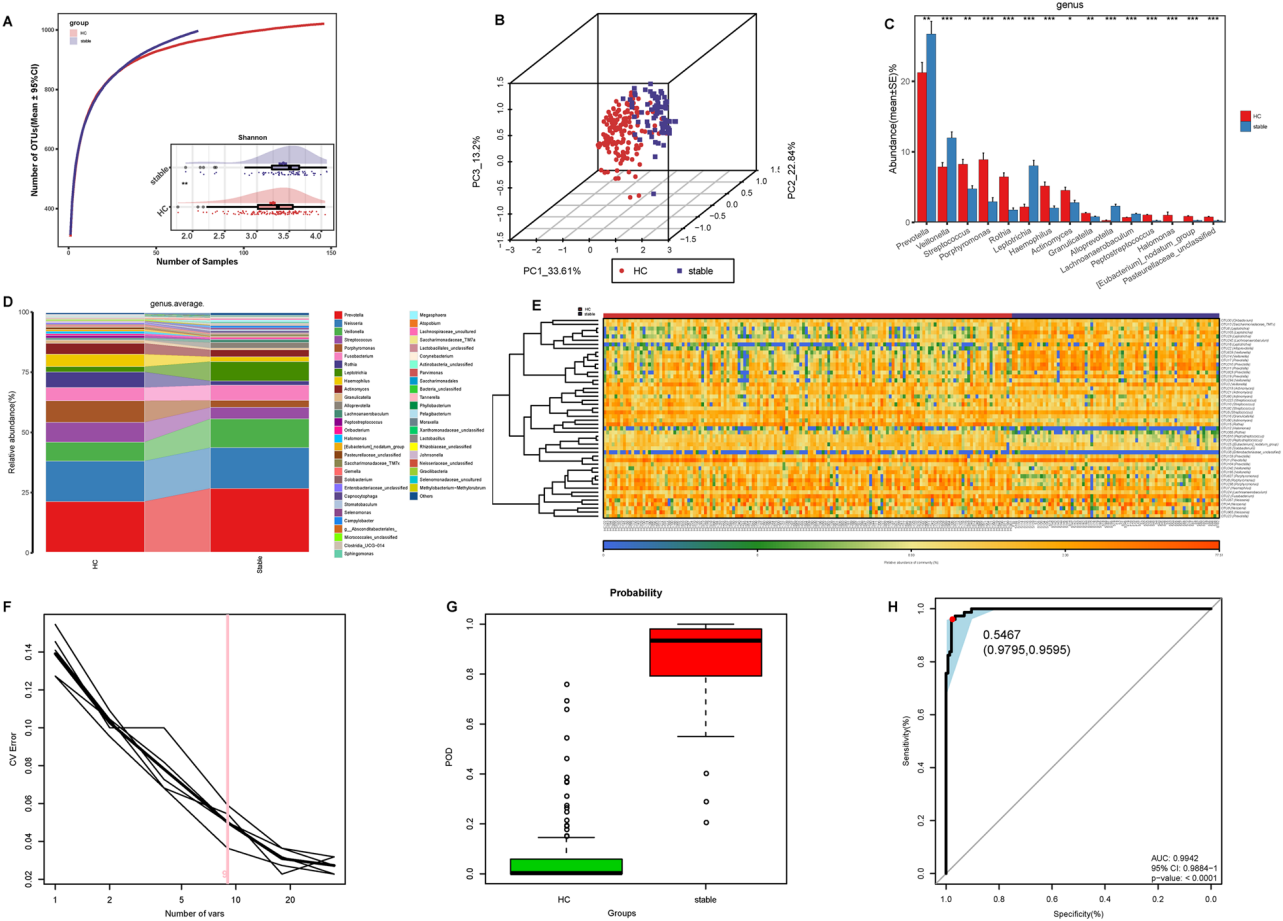
Alterations of oral microbiota in posttreatment stable SLE. **A** Rarefaction analysis between the number of samples and the number of OTUs. As the number of samples increased, the number of OTUs approached saturation in both groups, and the number of OTUs was significantly increased in SLE (n = 73) versus HC (n = 146). As measured by the Shannon index, the oral microbial diversity was significantly increased in SLE (n = 73) versus HC (n = 146) (p < 0.01). **B** The PCoA of oral microbial community for posttreatment stable SLE (n = 73) and HC (n = 146) in the unweighted Unifrac plot from PC1, PC2 and PC3 (33.61%, 22.84% and 13.2%). **C** Compared with HC, 21 genera were significantly increased, and 21 genera were significantly decreased in posttreatment stable SLE. **D** The average composition and relative abundance of the bacterial community in the posttreatment stable SLE (n = 73) and HC (n = 146) at the genus level. **E** Heatmap of the relative abundances of differential OTUs for each sample in the posttreatment stable SLE (n = 73) and HC (n = 146). The heatmap showed that 22 OTUs were enriched in SLE, and 28 OTUs were enriched in HC. **F** Nine OTUs were selected as the optimal markers set random forest model. **G** The POD value was significantly higher in SLE (n = 73) compared with that in HC (n = 146). **H** The POD value achieved an AUC of 0.9942 (95% CI 0.9884–1) between the posttreatment stable SLE (n = 73) and HC (n = 146) (p < 0.0001). *HC* healthy controls; stable, posttreatment stable SLE; *OUT* operational taxonomic unit; *CV Error* the cross-validation error, *POD* probability of disease, *CI* confidence interval, *AUC* area under the curve

To evaluate the oral microbiota features of patients with SLE in posttreatment stable condition, we further collected tongue-coating samples of 73 posttreatment SLE with SLEDAI = 0, and compared the oral microbiota features with those of 146 HC. Oral microbial diversity showed significant increase in posttreatment stable SLE compared to HC (Fig. 7A). The beta diversity analysis showed an obvious difference in the microbiome space community between the two groups (Fig. 7B). At the genus level, the average composition and relative abundance of the oral microbiota in both groups were shown in Fig. 7D. Increased abundance in 21 bacteria and decreased abundance in 21 bacteria were observed in stable SLE versus HC (Fig. 7C, Additional file 2: Data S22). The heatmap showing the top 50 different OTUs revealed that 22 OTUs enrichment was seen in SLE, and 28 OTUs enrichment was seen in HC (Fig. 7E). A random forest classifier model was established between 73 SLE and 146 HC using nine OTUs to identify the diagnostic potential of oral microbial markers for posttreatment stable SLE (Fig. 7F). The POD estimated value in SLE was considerably higher than in HC (p < 0.05), (Fig. 7G, Additional file 2: Data S23), with an AUC of 0.9942 (95% CI 0.9884–1, p < 0.0001) (Fig. 7H), which indicated that the oral microbial marker-based classifier model had powerful diagnostic potential for distinguishing posttreatment stable SLE from HC.

## Discussion

During the study, we comprehensively characterized the oral microbiota of treatment-naïve SLE cases in a large sample of the Chinese population by 16S rRNA MiSeq sequencing. We identified the crucial bacterial and microbial functions related to SLE and investigated the correlativity between oral microbiota and clinically collected data of SLE. Notably, we constructed a microbial classifier that demonstrated strong diagnostic efficacy in distinguishing SLE from HC and achieved cross-regional validation. In addition, we profiled the oral microbiome in SLE with different disease activities. Finally, we analyzed the oral microbiota alterations in posttreatment SLE and identified the optimal microbiota biomarkers. These findings suggested that oral microbiota dysbiosis may be involved in SLE development and recovery, and the microbiota classifier may have potential as a non-invasive biomarker that can be valuable in SLE diagnosis.

We found that the oral microbiome was dysbiosis in SLE, which displayed increased oral microbiome diversity and an altered bacterial community. One study showed that SLE patients had higher oral microbiome diversity than HC in subjects without periodontal disease [27]. Another study observed a difference in oral microbiome diversity between SLE and primary Sjogren’s Syndrome, and SLE had an increased oral microbiome diversity compared with primary Sjogren’s Syndrome [26]. Li et al. found that oral microbiome diversity was decreased in SLE patients compared to HC [25]. Yet, the study sample was small and enrolled both treatment-naïve and posttreatment SLE patients, which may weaken the reliability of the findings. Moreover, diet and environment may partly contribute to the different results between different studies. Additionally, we found that the taxa composition, crucial bacteria, and predicted microbial functions of the oral microbiome were different between SLE and HC. Zhang et al. found that the oral microbiota bacterial community and richness were different between RA and HC [21]^.^ Recently, we characterized the oral microbiome of patients with COVID-19 and found that the phylogenetic profiles, crucial bacteria and predicted microbial functions of COVID-19 differed significantly from those of HC [24]. In addition, one study found that in diabetic mice, the disturbed oral microbiota possesses enhanced pathogenicity [22]. Importantly, studies have shown that disturbance of the gut microbiota may trigger and promote the autoimmunity and inflammatory response in SLE by various pathways [8–14]^.^ These findings suggested that SLE owns its unique oral microbiome and the altered oral microbiota may be implicated in the initiation and pathogenesis of SLE.

The specific oral microbiome can be used as a diagnostic marker, and this has been studied in many diseases. Zhang et al. developed a diagnostic model in RA using the oral microbiota and verified its powerful diagnostic efficacy in an independent cohort [21]. Flemer established an oral microbiota-based diagnostic model that showed high specificity in identifying colorectal cancer from HC [23]. In this study, we developed and validated an SLE classifier based on two optimal OTUs of the oral microbiome. We calculated the POD of each sample in the derivation, independent validation, and cross-regional validation groups, which yielded AUCs of 0.9926, 0.9809, and 0.9851, respectively. This study is the first to successfully conduct an oral microbiota-based SLE classifier and achieve independent validation and cross-regional validation, suggesting that the oral microbiota could potentially serve as a non-invasive diagnostic method for SLE.

In addition, further analyses were performed to establish the correlation between oral microbiota and clinical indices in SLE. This analysis revealed a close association between the oral microbiota and seven clinical indices. As shown in our previous study, the abundance of specific bacteria in the gut microbiota was related to clinical indicators in chronic renal disease [32]. Ma et al. also found a strong correlation between gut microbiota and serum indices in autoimmune hepatitis [33]. Another study demonstrated a significant correlation between the variation in gut microbiota and clinical data in SLE [17]. Notably, one important finding of our study was that the oral microbiome characteristics differed in different SLE disease activities. As disease activity increased in SLE, the abundance of Abiotrophia and Lactobacillales_P5D1-392 increased, and the abundance of Phyllobacterium and Micrococcaceae_unclassified decreased. These results suggested that oral microbiota alteration may be involved in SLE progression and acceleration. Currently, studies have found that dysbiosis of gut microbiota could play a role in SLE progression, and disease activity can be partly relieved by probiotics, diet intervention, and antibiotics [12]. We hypothesized that interventions targeting the oral microbiota may relieve disease activity and become a therapeutic approach for SLE in the future.

It has been observed that the microbiota altered as disease recovery. One study found that the perturbed oral microbiota partly returned to normal in RA after treatment [21]. Similar results have been observed in periodontitis [34]. Our previous study found that the oral microbiota was related to disease recovery in COVID-19 [24]. During this research, the features of oral microbiota of SLE patients in stable posttreatment conditions were evaluated. The results showed that although patients with SLE achieved remission with neither clinical nor serological activity following treatment, the oral microbiota was dysbiosis and differed from that of HC. Moreover, we established a diagnostic model based on oral microbiota that could differentiate posttreatment stable SLE patients from HC. The results suggested that the oral microbiota may be involved in the disease recovery of SLE.

It is worth doing note that antioxidants may have a potential therapeutic effect in SLE patients. Studies have shown that T cells in lupus mice and SLE patients are continuously activated and therefore have hyperpolarized mitochondria compared to T cells in healthy mice or healthy individuals, which can lead to increased production of reactive oxygen species [35, 36]. In addition, the levels of glutathione and cysteine in serum and peripheral blood individual nuclei of SLE patients are lower than with healthy individuals, suggesting the presence of oxidative stress [37]. It has been shown that antioxidants have specific pharmacological effects in autoimmune diseases [38, 39].

## Conclusion

In summary, we firstly profiled the characteristic alterations of the oral (tongue) microbiota in SLE across a large cohort, identified the crucial bacteria and predicted the specific microbial functions related to SLE. Additionally, we revealed that the oral microbiota was correlated with clinical indicators in SLE and that the oral microbiota was altered as disease activity increased. These results suggested that oral microbiota dysbiosis may contribute to the development of SLE. In addition, we are the first to develop a microbial-based SLE classifier and validate its diagnostic efficiency in patients from multiple areas around China, providing a non-invasive diagnostic tool for SLE. Furthermore, we characterized the oral microbiota in posttreatment SLE patients under stable conditions, which suggested that the oral microbiota may has a relation to disease recovery in SLE. However, the outcomes need validation in multicenter cohorts of individuals of different ethnicities. In the end, these findings suggested that the oral microbiota may be a therapeutic target for exploring new treatment approaches for SLE in the future.

## Contribution to the field

Systemic lupus erythematosus (SLE) is a chronic autoimmune disease of unknown etiology and mechanism. It has been shown that oral microbiota biomarkers could be served as a non-invasive diagnostic tool for many diseases, however, it is unclear whether oral microbiome could discriminate SLE from healthy controls. Compared with previous studies, we firstly analyzed characteristic alterations in the oral (tongue) microbiome of SLE in a large cohort, identified key bacteria associated with SLE and predicted specific microbial functions, and developed a first non-invasive microbial-based model for diagnosing SLE, validating its diagnostic efficiency for the first time in patients from multiple regions of China. Furthermore, we characterized the oral microbiome of treated SLE patients under stable conditions, which suggests that the oral microbiome may be associated with disease recovery in SLE. Our study suggests that oral microbiota dysbiosis may contribute to the development and progression of SLE. In addition, our study lays the foundation for future targeted oral microecological treatment of SLE.

## Supplementary Information

**Additional file 1: Figure S1–17** and legends.

**Additional file 2:** Supplementary Data **S1–23**.

## Data Availability

The datasets supporting the conclusions of this article are available in the Sequence Read Archive database repository, https://www.ncbi.nlm.nih.gov/sra/.
