# Supplementary material for: Alterations in the human oral microbiota in systemic lupus erythematosus
Source: J Transl Med. 2023 Feb 8;21:95. doi: 10.1186/s12967-023-03892-3 (PMC9905765; doi:10.1186/s12967-023-03892-3)
Supplement: Supplementary file 1 — Additional file 1: Figure S1–17 and legends. [file 12967_2023_3892_MOESM1_ESM.pdf]

Additional file 1 Figure S1-17 and legends

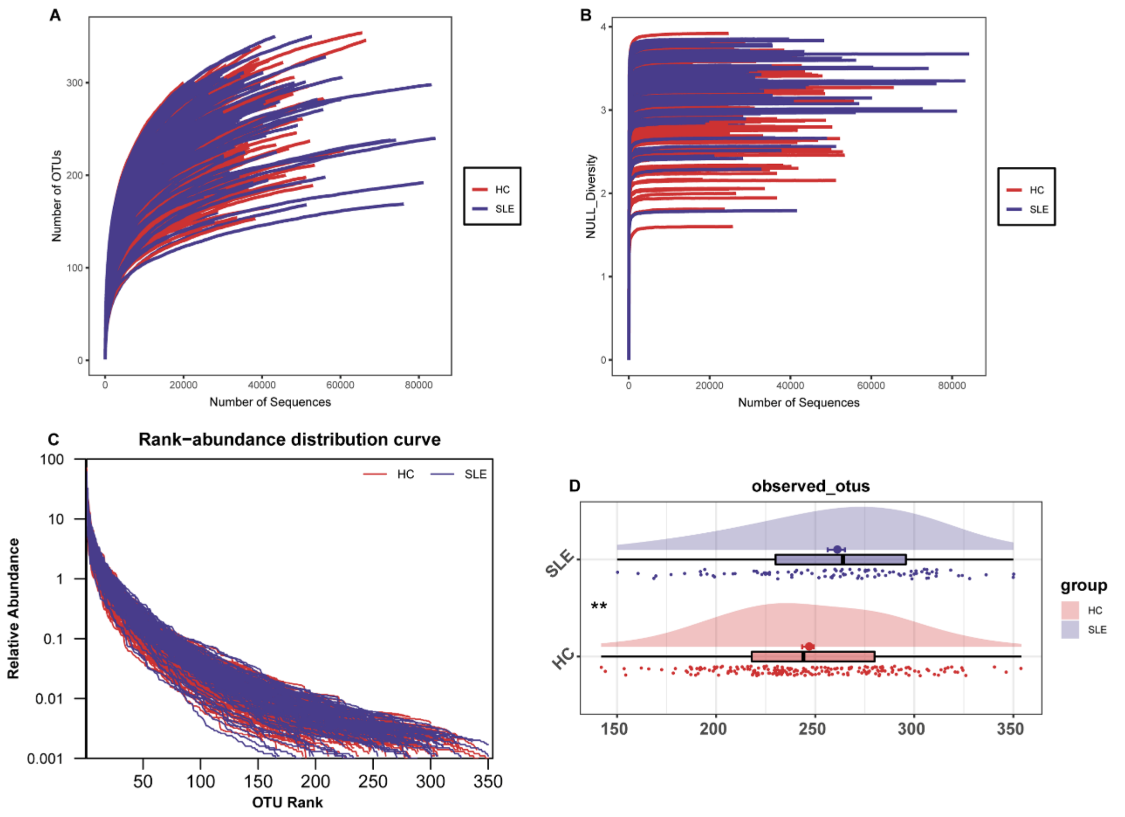

Figure S1. Results of rarefaction analysis.

A. A rarefaction curve between the number of OTUs and the number of sequences in SLE (n=100) and HC (n=200).

B. A Shannon-wiener curve between the number of sequences and the null diversity in SLE (n=100) and HC (n=200).

C. A rank-abundance distribution curve for the OTUs of SLE (n=100) and HC (n=200).

D. In the derivation cohort, the observed OTUs were significantly increased in SLE (n=100) compared with that in HC (n=200).

SLE, systemic lupus erythematosus; HCs, healthy control; OTU, operational taxonomic unit. **, p<0.01.

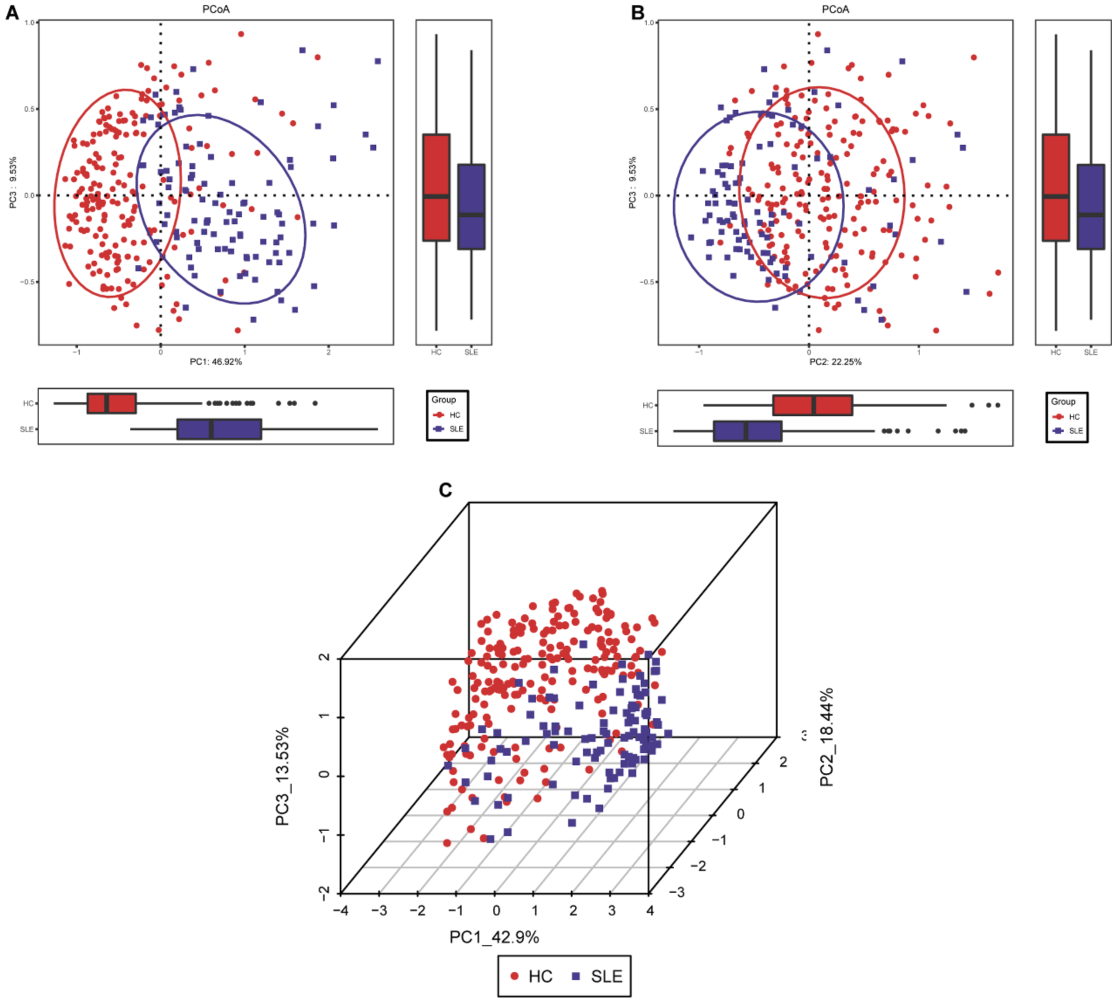

Figure S2. OTUs distribution between the two groups

A. The PCoA of oral microbial community for SLE (n=100) and HC (n=200) in the unweighted Unifrac plot from PC1 and PC3 (46.92% and 9.53%).

B. The PCoA of oral microbial community for SLE (n=100) and HC (n=200) in the unweighted Unifrac plot from PC2 and PC3 (22.25% and 9.53%).

C. The PCoA of oral microbial community for SLE (n=100) and HC (n=200) in the unweighted Unifrac plot from PC1, PC2 and PC3 (42.9%, 18.44% and 13.53%).

HC, healthy controls; SLE, systemic lupus erythematosus; PCoA, principal coordinate analysis.

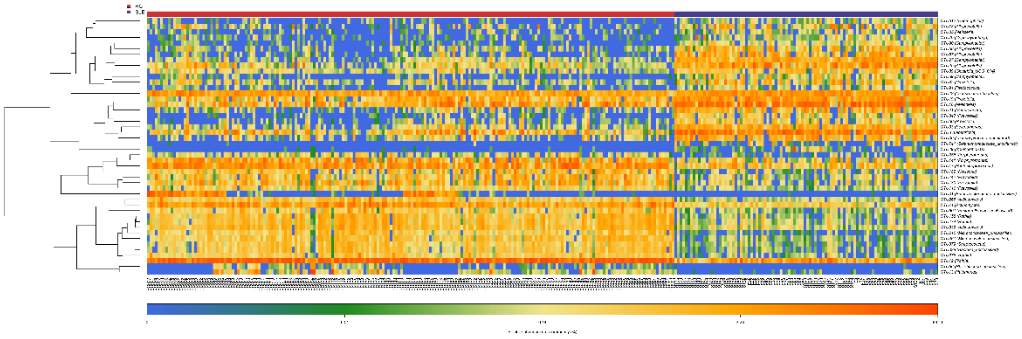

Figure S3. The relative abundance and distribution of 46 OUTs between the two groups

A heatmap showed the relative abundance and distribution of the key 46 OUTs between the two groups. Notably, 24 OUTs were significantly enriched in SLE, and 22 OTUs were significantly enriched in HC.

HC, healthy controls; SLE, systemic lupus erythematosus; OTU, operational taxonomic unit.

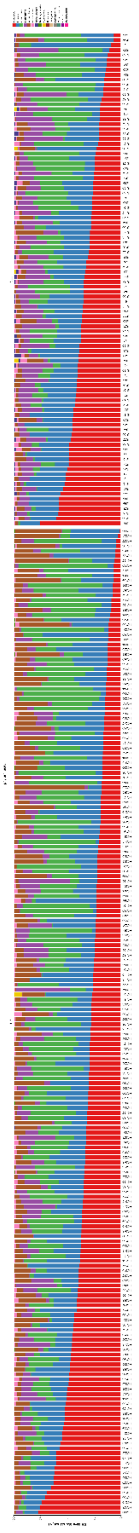

Figure S4. The composition and abundance of bacterial community in each sample of the derivation cohort at the phylum level (200 HC and 100 SLE).

HC, healthy controls; SLE, systemic lupus erythematosus.

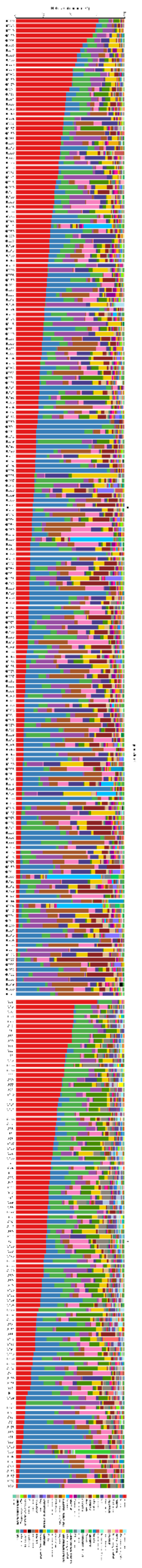

Figure S5. The composition and abundance of bacterial community in each sample of the derivation cohort at the genus level (200 HC and 100 SLE).

HC, healthy controls; SLE, systemic lupus erythematosus.

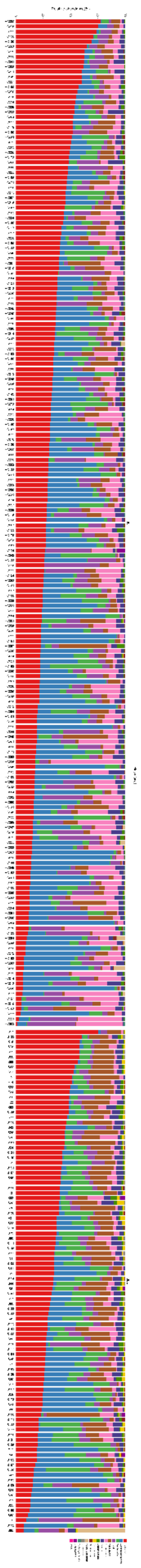

Figure S6. The composition and abundance of bacterial community in each sample of the derivation cohort at the class level (200 HC and 100 SLE).

HC, healthy controls; SLE, systemic lupus erythematosus.

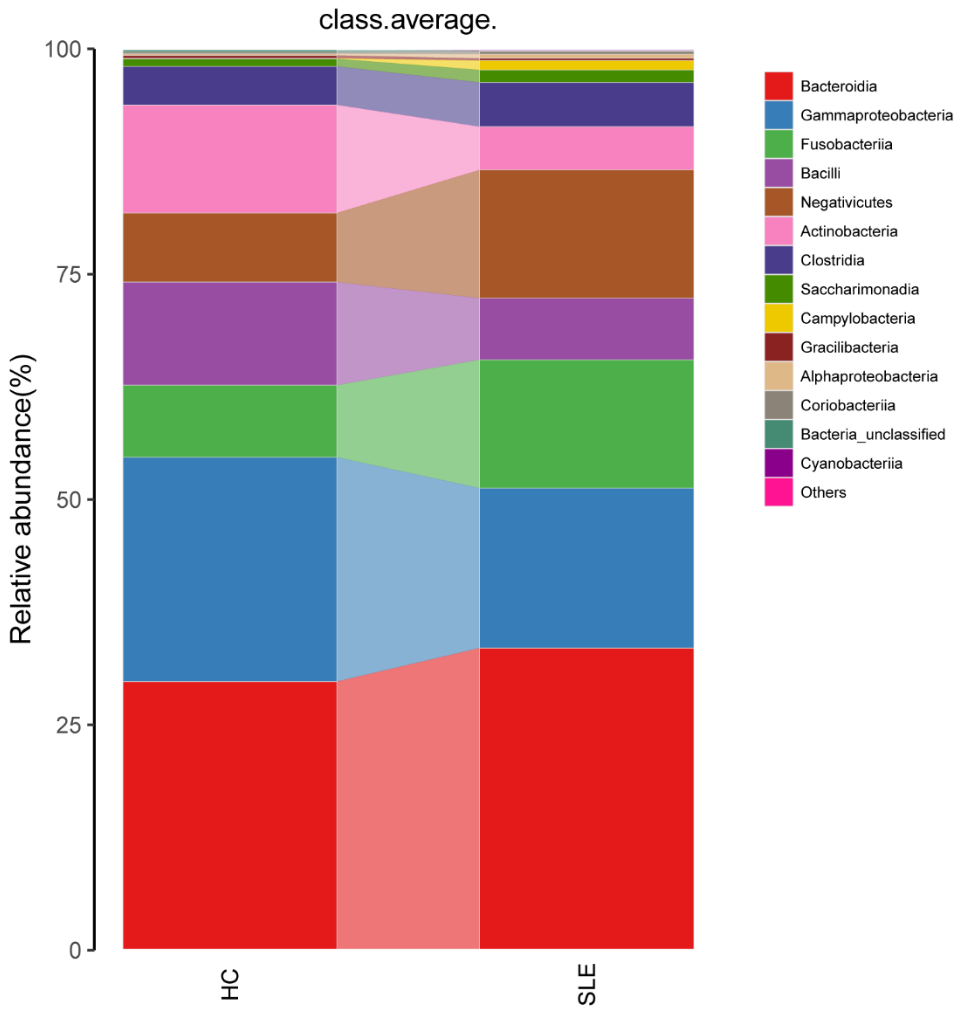

Figure S7. The average composition and relative abundance of the bacterial community at the class level in both groups of the derivation cohort (200 HC and 100 SLE).

HC, healthy controls; SLE, systemic lupus erythematosus.

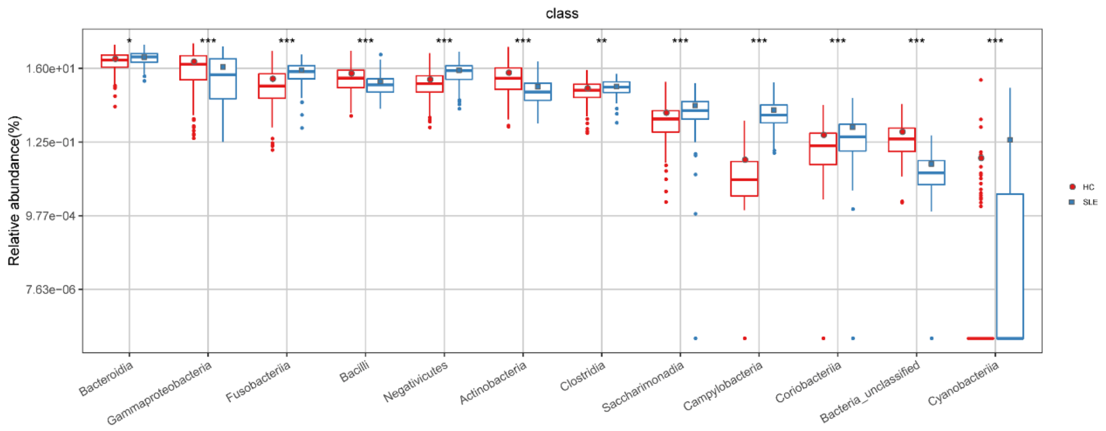

Figure S8. At the class level, eight bacterial populations were significantly enriched, and four bacterial populations were significantly reduced in SLE (n=100) versus HC (n=200).

*, p<0.05, **, p<0.01, ***, p<0.001; HC, healthy controls; SLE, systemic lupus erythematosus.

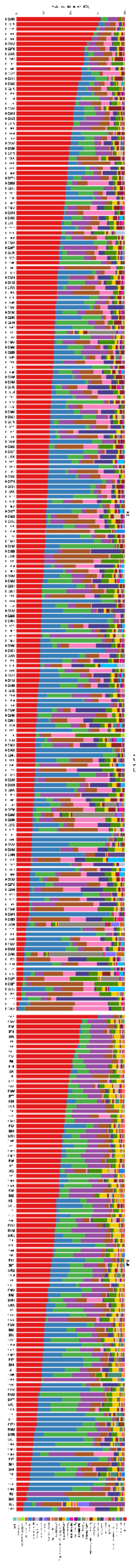

Figure S9. The composition and abundance of bacterial community in each sample of the derivation cohort at the order level (200 HC and 100 SLE).

HC, healthy controls; SLE, systemic lupus erythematosus.

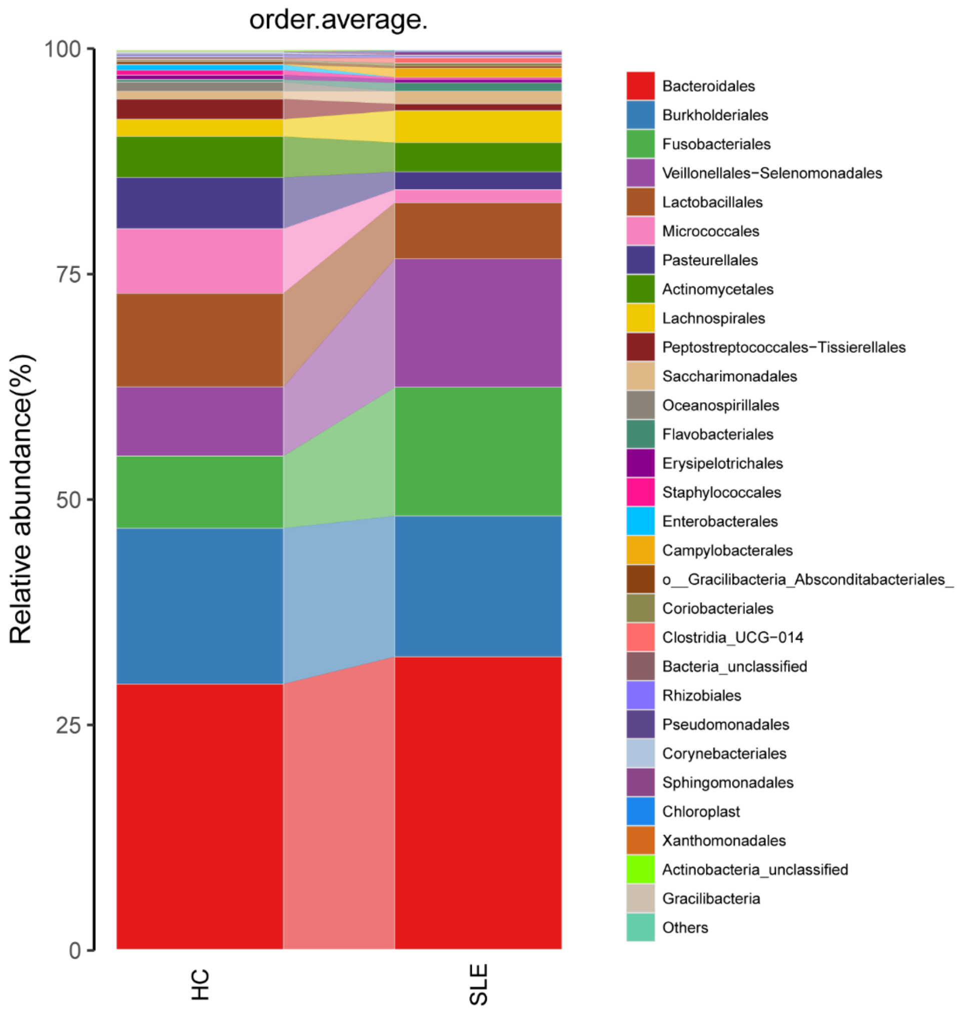

Figure S10. The average composition and relative abundance of the bacterial community at the order level in both groups of the derivation cohort ((200 HC and 100 SLE)).

HC, healthy controls; SLE, systemic lupus erythematosus.

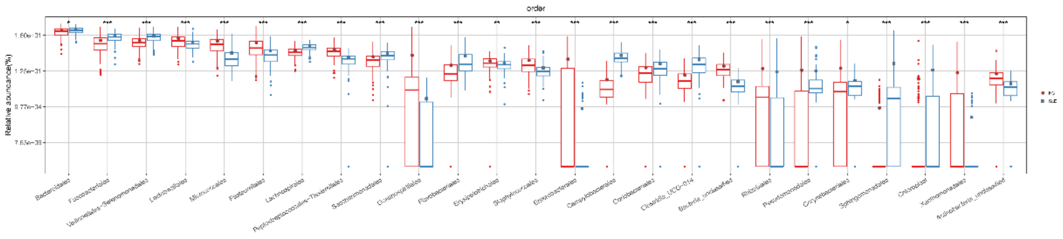

Figure S11. At the order level, 13 bacterial populations were significantly enriched, and 12 bacterial populations were significantly reduced in SLE (n=100) versus HC (n=200).

*, p<0.05; **, p<0.01; ***, p<0.001; HC, healthy controls; SLE, systemic lupus erythematosus.

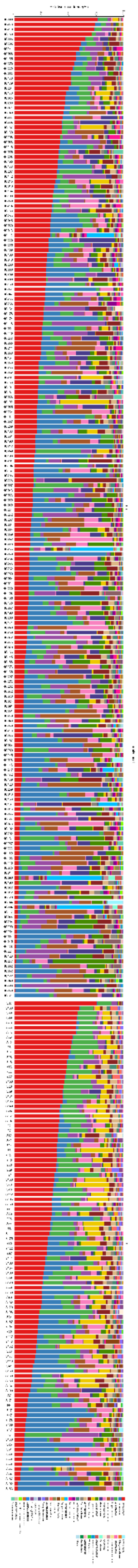

Figure S12. The composition and abundance of bacterial community in each sample of the derivation cohort at the family level (200 HC and 100 SLE).

HC, healthy controls; SLE, systemic lupus erythematosus.

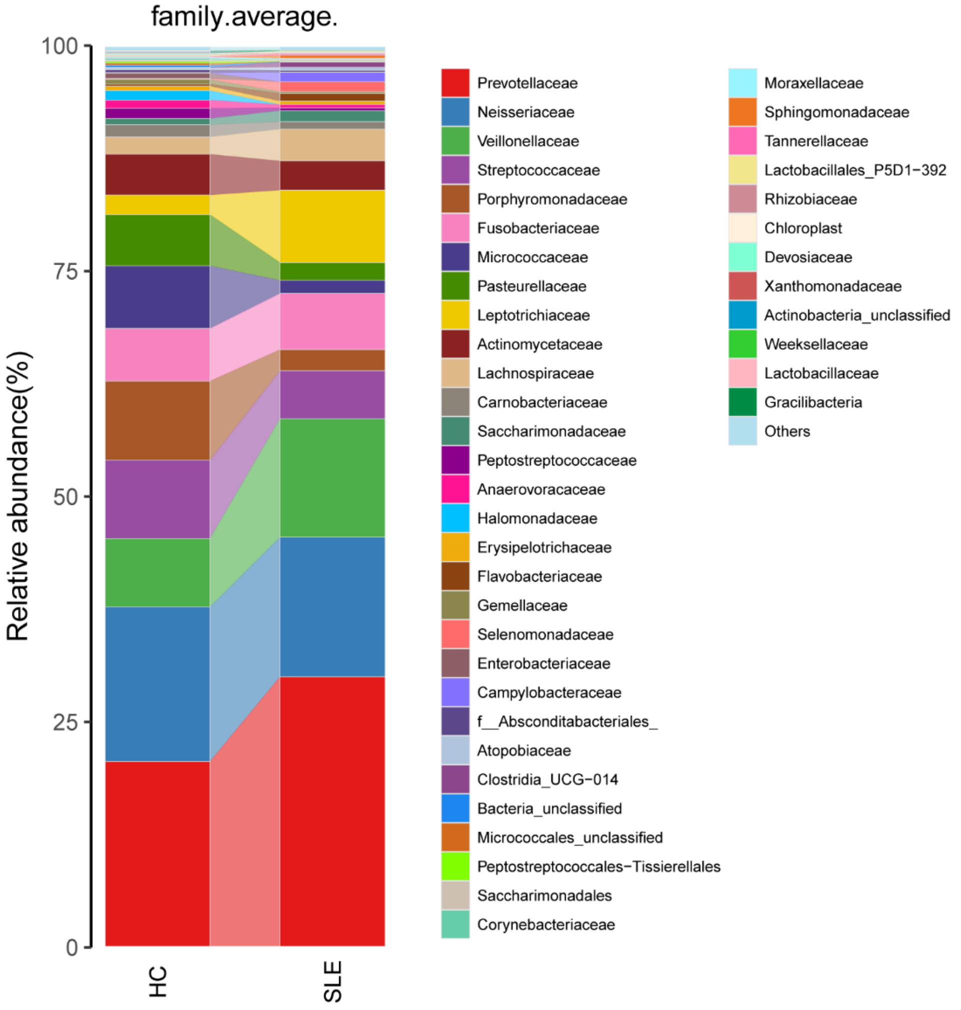

Figure S13. The average composition and relative abundance of the bacterial community at the family level in both groups of the derivation cohort ((200 HC and 100 SLE)).

HC, healthy controls; SLE, systemic lupus erythematosus.

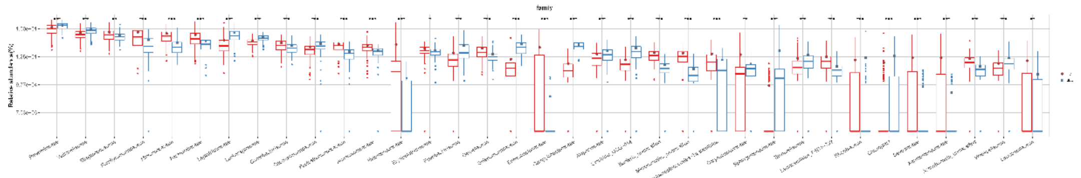

Figure S14. At the family level, 17 bacterial populations were significantly enriched, and 18 bacterial populations were significantly reduced in SLE (n=100) versus HC (n=200).

*, p<0.05; **, p<0.01; ***, p<0.001; HC, healthy controls; SLE, systemic lupus erythematosus.

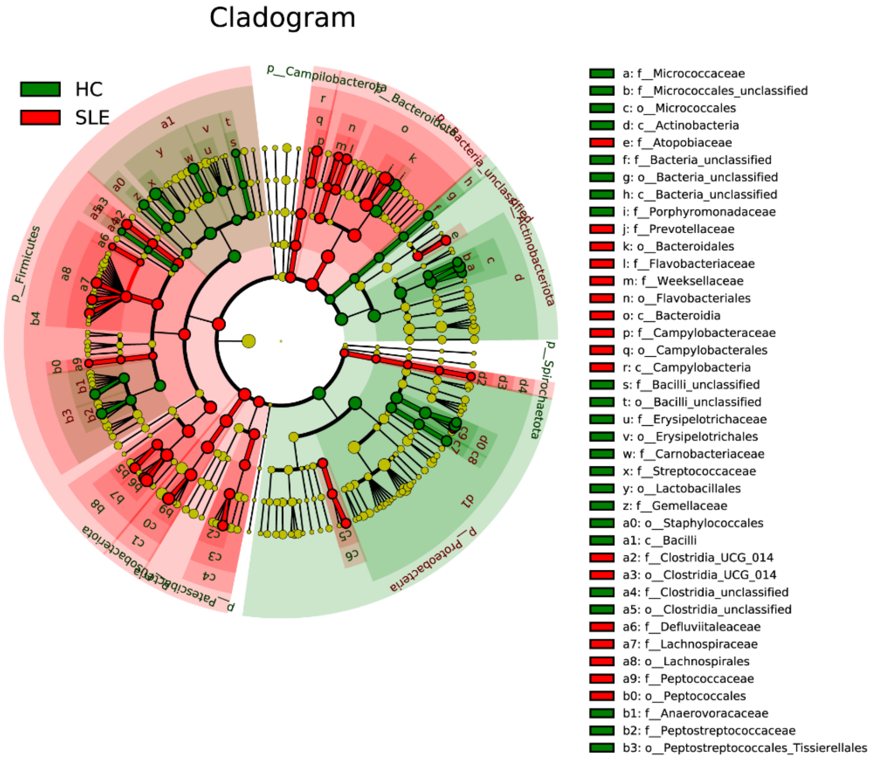

Figure S15. The phylogenetic profiles of the specific bacterial taxa and predominant bacteria associated with SLE (n=100) and HC (n=200) using the LEfSe method.

HC, healthy controls; SLE, systemic lupus erythematosus; LEfSe, linear discriminant analysis effect size.

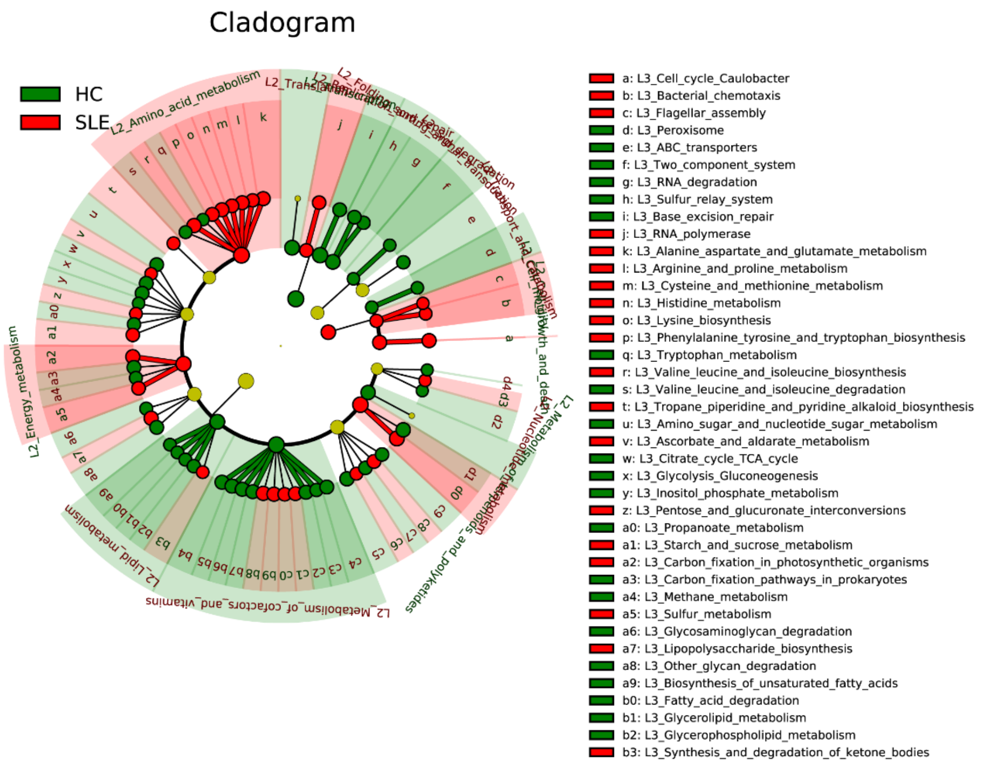

Figure S16. A cladogram showed the oral microbial community function profiles of SLE (n=100) and HC (n=200) and their predominant microbial functions.

HC, healthy controls; SLE, systemic lupus erythematosus.

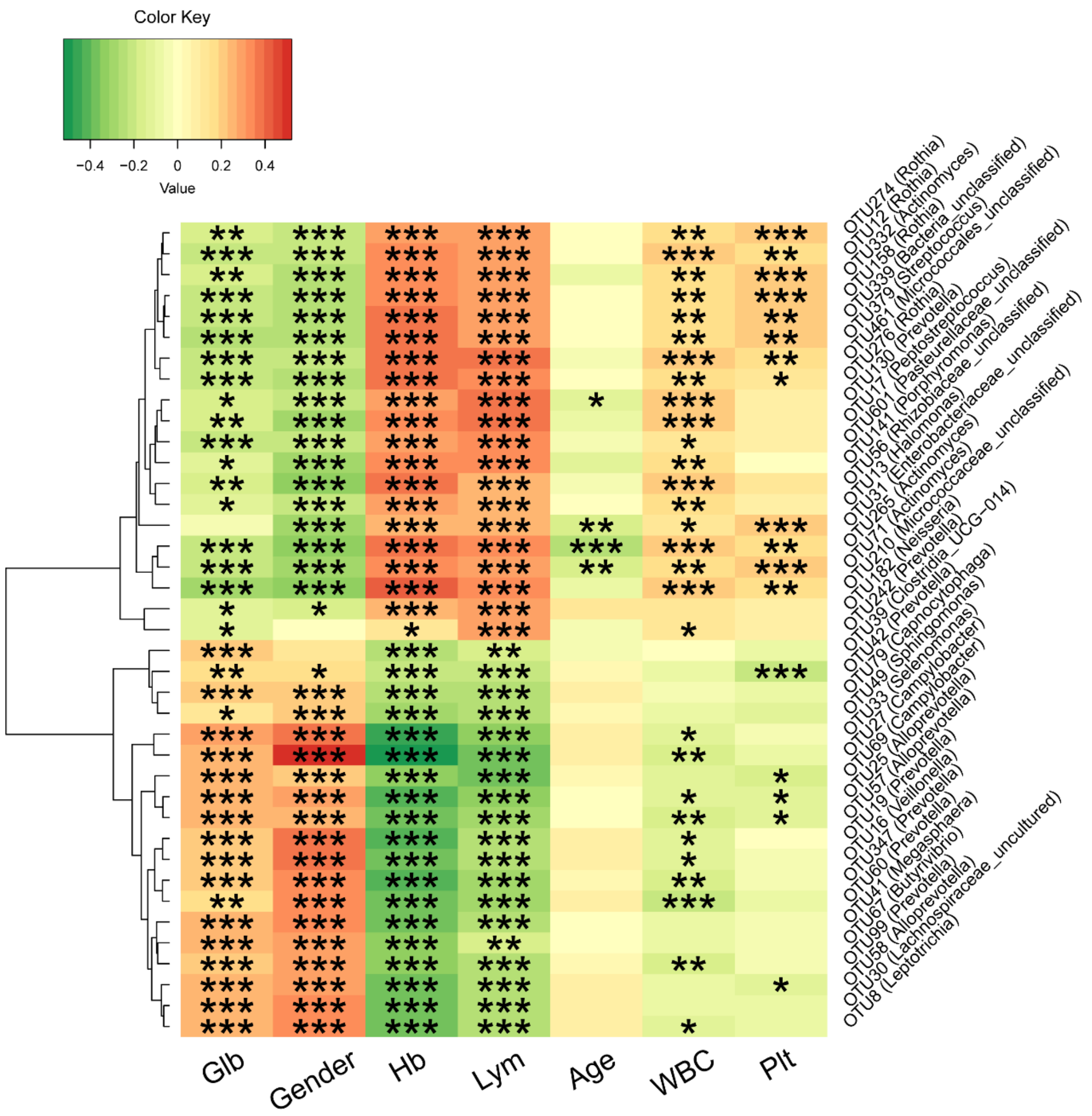

Figure S17. Heatmap showing the partial Spearman's correlation coefficients among 39 OTUs and seven clinical indicators of SLE (n=100).

OTU, operational taxonomic unit; WBC, white blood cell; Hb, hemoglobulin; Plt, platelet count; Lym, lymphocyte count; Glb, globulin.
