# Supplementary material for: Alterations in the human oral microbiota in systemic lupus erythematosus
Source: J Transl Med. 2023 Feb 8;21:95. doi: 10.1186/s12967-023-03892-3 (PMC9905765; doi:10.1186/s12967-023-03892-3)
Supplement: Supplementary file 2 — Additional file 2: Supplementary Data S1–23. [file 12967_2023_3892_MOESM2_ESM.zip › 12967_2023_3892_MOESM2_ESM/Data S1-S23 and legends/Additional file 3.docx]

**Additional file 2 Data S1-23 legends**

**Additional file 2 Data S1.** The detailed values of oral microbial diversity index and observed OTUs in the derivation cohort (200 healthy controls and 100 SLE).

**Additional file 2 Data S2.** The relative abundance and distribution of the key 46 OUTs in the derivation cohort (200 healthy controls and 100 SLE).

**Additional file 2 Data S3.** The composition and abundance of bacterial community at the phylum level in each sample in the derivation cohort (200 healthy controls and 100 SLE).

**Additional file 2 Data S4.** The composition and abundance of bacterial community at the genus level in each sample in the derivation cohort (200 healthy controls and 100 SLE).

**Additional file 2 Data S5.** The different degree at the phylum level (p value) between the two groups in the derivation cohort (200 healthy controls and 100 SLE).

**Additional file 2 Data S6.** The different degree at the genus level (p value) between the two groups in the derivation cohort (200 healthy controls and 100 SLE).

**Additional file 2 Data S7.** The different degree at the class level (p value) between the two groups in the derivation cohort (200 healthy controls and 100 SLE).

**Additional file 2 Data S8.** The different degree at the order level (p value) between the two groups in the derivation cohort (200 healthy controls and 100 SLE).

**Additional file 2 Data S9.** The different degree at the family level (p value) between the two groups in the derivation cohort (200 healthy controls and 100 SLE).

**Additional file 2 Data S10.** The specific bacterial taxa and predominant bacteria associated with SLE (n=100) and HC (n=200) by LEfSe method.

**Additional file 2 Data S11.** The corresponding LDA value and p value of the significantly different genera in the derivation cohort (200 healthy controls and 100 SLE).

**Additional file 2 Data S12.** The corresponding LDA value and p value of microbial community gene function for samples in the derivation cohort (200 healthy controls and 100 SLE).

**Additional file 2 Data S13.** The p value of Spearman’s correlation analysis between oral microbiome and clinical data of SLE in the derivation cohort (200 healthy controls and 100 SLE).

**Additional file 2 Data S14.** The corresponding output value of each optimal microbial marker in the derivation cohort by random forest classifier model (200 healthy controls and 100 SLE).

**Additional file 2 Data S15.** The corresponding POD value of each sample in the derivation cohort (200 healthy controls and 100 SLE).

**Additional file 2 Data S16.** The corresponding output value of each optimal microbial marker in the independent validation cohort by random forest classifier model (80 healthy controls and 40 SLE from Zhengzhou, China).

**Additional file 2 Data S17.** The corresponding output value of each optimal microbial marker in the cross-reginal validation cohort by random forest classifier model (80 healthy controls and 42 SLE from Haikou, China).

**Additional file 2 Data S18.** The corresponding POD value of each sample in the independent validation cohort (80 healthy controls and 40 SLE from Haikou, China).

**Additional file 2 Data S19.** The corresponding POD value of each sample in the cross-reginal validation cohort by random forest classifier model (80 healthy controls and 42 SLE from Haikou, China).

**Additional file 2 Data S20.** The different degree of genus level (p value) among the mild (n=68), moderate (n=58), and severe (n=14) disease activity groups.

**Additional file 2 Data S21.** The bacterial abundance and composition of each sample in mild (n=68), moderate (n=58), and severe (n=14) disease activity groups.

**Additional file 2 Data S22.** The different degree at the genus level (p value) between the posttreatment stable SLE (n=73) and healthy controls (n=146).

**Additional file 2 Data S23.** The corresponding POD value of each sample in the posttreatment stable SLE (n=73) and healthy controls (n=146).
